# What makes patients tick? Vaccine preferences against tick-borne encephalitis in four European countries

**DOI:** 10.1186/s12879-024-10045-4

**Published:** 2024-10-13

**Authors:** Charlotta Zacharias, Ralph Torgler, Jennifer Cummins

**Affiliations:** 1 VaccinDirekt Sverige AB, Slussplan 7, Stockholm, 111 30 Sweden; 2 Bavarian Nordic Switzerland AG, Grafenauweg 8, Zug, CH-6301 Switzerland

**Keywords:** Tick-borne encephalitis, Tick-borne encephalitis vaccine, Patient preference, Discrete-choice, Patient support, Vaccine attribute, Vaccine booster interval, Patient choice

## Abstract

**Background:**

We explored vaccine motivation and preferences for tick-borne encephalitis (TBE) vaccine attributes among participants in TBE-endemic countries in Europe.

**Methods:**

An online survey was conducted among the general public in Austria, Germany, Switzerland, and Sweden. Participants were ≥ 18 years old, open to receiving vaccines, and living in, or regularly traveling to, TBE-endemic regions in the aforementioned countries. Participants were asked about their general vaccine knowledge and motivations for vaccination, before rating the importance of TBE vaccine attributes, such as efficacy, safety, dosing schedule, and booster interval. Thereafter, participants were shown three hypothetical TBE vaccine profiles with different combinations of attributes. Assuming equal efficacy and safety, participants were asked to select their preferred profile from 12 screens as part of a discrete-choice conjoint analysis. Utility scores were calculated to show the importance of each attribute. Data are presented for the overall survey group and by age and gender, using t-tests to compare means.

**Results:**

For 73% of participants (*n* = 1003/1379), self-protection was among the top three reasons to get vaccinated. Disease severity, protection of children or family, and advice or recommendation from a doctor/healthcare professional (HCP) were top three reasons for over half of participants. The majority (58–69%) agreed or strongly agreed that they trust their doctor/HCP on the subject of vaccines, they rely on their doctor/HCP’s vaccine knowledge, and they prefer their doctor/HCP to make recommendations on which vaccines they or their families should take. Efficacy and safety were the most important standalone TBE vaccine attributes; however, among TBE vaccine profiles including 3-, 5- and 10-year booster intervals, the 10-year booster interval was the most influential attribute level when choosing a preferred vaccine profile (utility score: 0.58 [standard error: 0.01]). Differences in motivators and preferences were observed between age and gender subgroups.

**Conclusion:**

The high level of doctor/HCP reliance highlights the key role doctors/HCPs play in influencing vaccine decision-making. Booster interval was the biggest driver of choice when selecting a hypothetical TBE vaccine profile, with the strongest preference for a 10-year booster interval. These findings could be used to inform TBE vaccination recommendations and in the further development of TBE vaccines.

**Supplementary Information:**

The online version contains supplementary material available at 10.1186/s12879-024-10045-4.

## Background

Tick-borne encephalitis (TBE), caused by the TBE virus (TBEV), is a vector-borne neurological disease affecting humans and animals [1]. Transmission predominantly occurs via a bite from an infected *Ixodes* tick [2] in outdoor and rural environments [3]. TBE is considered endemic in 27 countries throughout Europe [4, 5], though endemnicity within each country can vary from region to region [4]. There are a reported 5–12,000 cases per year [6] in Europe; however, mild or asymptomatic cases are likely to be under-reported [5, 7, 8], and country-to-country reporting standards vary substantially [2]. Recent reports suggest that the number of TBE cases has significantly increased across Europe in the last five years alone [9–11]. Potential reasons for this are complex and multifactorial, though greater time spent outdoors in tick-infested areas, increased exposure through minimally processed and locally produced food, and climatic changes favoring increased vector activity and spread are likely to be driving factors [12].

Historically, there were thought to be three main viral strains of TBEV: the European, Siberian, and Far Eastern strains, named according to their geographical distribution [1]. However, all three TBE variants are now commonly found in the Baltic states, while European and Siberian variants are present in Finland, indicating the spread of the disease in Europe [2, 13, 14]. Symptoms of TBE include non-specific, mild flu-like symptoms, though patients can also be asymptomatic [15]. While the majority of people will make a full recovery, others may experience a second phase [6], characterized by central nervous system involvement, such as meningitis or encephalitis, which may lead to long-term neurological sequelae and death in some cases [7]. Symptom severity, secondary neurological complications, and mortality rate can vary substantially depending on strain [15]. The European strain, for example, is generally associated with milder symptoms and a mortality rate of approximately 2% [15]. Up to a third of patients with the European strain will experience a second phase of infection, and around 10% may experience long-term neurological sequelae [15]. Conversely, the Far Eastern strain is considered to be the most severe, with mortality rates of up to 35% [15].

Currently, there are no specific antiviral treatments for TBE. Two preventative vaccines, Encepur and FSME-IMMUN (TicoVac), are approved for use throughout Europe [16]. Evidence suggests that receiving three doses of either vaccination, as recommended for the primary immunization schedule, results in seroconversion rates of 90–98% [17–19]. Thereafter, protection may wane, particularly in individuals over 50 years old [17, 18]. Thus, current prescribing information suggests that individuals should receive a first booster within 1–3 years of completing the primary schedule, and every 3–5 years, thereafter, depending on age [17–19]; though dosing schedules and booster recommendations may vary from country to country for specific at-risk groups [20].

Reimbursement policies and public health recommendations for who should be vaccinated also vary substantially according to country-specific needs [21]. Austria, for example, recommends vaccination for the entire population, irrespective of risk, and conducts public health TBE awareness and vaccination campaigns each year [22, 23]. Conversely, countries such as Germany, Sweden and Switzerland recommend vaccination only for at-risk groups owing to age, occupation, lifestyle factors, or residency [22].

Initial uptake for the TBE vaccine is highly heterogeneous across Europe [24], though compliance with the primary immunization and booster schedules is generally low [23]. One possible explanation for this could be a lack of perceived risk and insufficient TBE knowledge among at-risk individuals living or working in endemic regions [2, 23]. In countries without a recommend-to-all approach for the TBE vaccine, uptake is at least partly reliant upon individuals either being able to self-identify as at-risk or receiving the correct guidance from their doctor/healthcare professional (HCP), indicating the need to continually raise public and doctor/HCP awareness of TBE across Europe. Limited access to vaccination clinics, cost, and undesirable vaccine-specific attributes, such as unfavorable dosing schedules or booster time intervals, may also play a role in insufficient TBE vaccine uptake [2].

Given the continual rise in TBE cases across Europe and the geographical spread of TBE variants, there is an urgent need to improve disease prevention. Increasing vaccine uptake, regularly monitoring and revising vaccine recommendations in relation to changing risk, and continuing to update the labels of TBE vaccines based on new data, are key to achieving this goal. Here, we explored TBE vaccine preferences and motivating behaviors among people living in TBE-endemic regions in Europe in order to highlight key opportunities for improvement in each of the aforementioned strategies.

## Materials and methods

### Study design

A two-part, online, quantitative survey was designed to explore vaccine preferences among people living in four TBE-endemic countries in Europe, using consumer panels provided by Dynata, a market research company. Participants were invited to take part in the survey via email, and the survey was hosted on the Omnisis online platform from 24/08/23–08/09/23. The first part of the survey assessed attitudes towards vaccines in general, and the second part explored the importance of specific TBE vaccine attributes on the selection of preferred hypothetical TBE vaccine profiles, using a discrete-choice conjoint analysis. The full questionnaire can be viewed in Supplementary File 1.

### Study participants

Study participants consisted of adult members of the general public from Austria, Germany, Sweden, or Switzerland. Eligible participants were required to be ≥ 18 years old, open to receiving vaccines, and living in, or regularly traveling to, TBE-endemic regions in the aforementioned countries. Stratified Sampling with Proportional Allocation was not used to select samples or to determine sample sizes from the five countries. However, to ensure that the final sample was as representative as possible of the at-risk population, or those making decisions on vaccination for members of their family, all respondents were asked a maximum of seven demographic screening questions to enable them to progress to the main survey. The screener contained minimum and maximum quotas for each country on age, gender, TBE vaccination status, parents with children < 18 years old, and employment status. Specifically, there was a requirement to have ≥ 15% of participants in each age category (18–30, 31–50, 51–65, > 65 years old), ≤ 65% in male or female gender categories (other genders were not subject to minimum or maximum quotas), ≤ 20% of participants vaccinated against TBE, ≥ 20% of participants with children less than 18 years old, and representation in all employment categories. Once each demographic quota was met, any further participants within that particular demographic did not progress to the main survey.

Before taking part, participants were informed that the survey was conducted in accordance with the European Society for Opinion and Market Research (ESOMAR) and the European Pharmaceutical Market Research Association (EMPHRA) codes of conduct regarding anonymity and confidentiality of data. All participants were asked to consent on this basis, before proceeding. As this study did not involve an intervention, a disease-state, or human tissue, and there was no possibility of harm, no prior ethical approval was necessary, as per the relevant guidelines [25, 26]*.* All participants were reimbursed pro-rata at fair market value based on the survey completion time.

### Survey development

Initial qualitative scoping interviews were conducted with eight participants (four from Germany and four from Switzerland), to help define the most important TBE vaccine attributes from a patient perspective to be included in the final survey. This sample included three parents with children under the age of 18 years old, for whom they arranged vaccinations.

The survey was then pretested through a series of online interviews with simultaneous screen-sharing with three English-speaking participants. This was done to ascertain participant comprehension of each vaccine attribute, as well as to assess the clarity of the questions and overall user experience of the survey. The final version of the survey, based on feedback received during these interviews, was developed in English and translated into German, French, and Swedish. Translated versions were reviewed by native speakers to ensure clarity and consistency.

### Quantitative survey

The first part of the survey took approximately 25 min to complete. Participants were asked a number of multiple-choice, Likert-scaled questions relating to their general vaccine knowledge, doctor/HCP reliance for vaccine advice or recommendations, and general vaccine preferences. Survey participants were also asked to select their top three motivating reasons for getting any vaccination from a pre-defined list of reasons.

### Discrete-choice conjoint analysis

The second part of the survey took approximately 10–15 min to complete. It consisted of questions pertaining to the stated preference for individual TBE vaccine attributes and, thereafter, the importance of each attribute in the context of other attributes when selecting a preferred hypothetical TBE vaccine profile. The list of attributes was generated from suggested TBE vaccine attributes that arose from the initial qualitative interviews as part of the survey development. These included efficacy, side effect frequency, side effect severity, dosing schedule, booster time interval, ability to switch TBE vaccine brands, cross-protection against other geographically distinct TBE strains, vaccine manufacturer country of origin (location of pharmaceutical company’s headquarters), environmentally friendly packaging, and where to access the vaccine. To ensure that participants sufficiently understood all of the attributes, they were provided with simple and visual explanations of each one before answering any questions (see Table 1 within Supplementary File 1).

Using a Likert-scaled response (1, not very important–7, very important), participants were first asked to state the importance of each attribute as a standalone attribute when considering TBE vaccination for themselves or a family member. A discrete-choice conjoint method [27] was then used to assess the importance of varying levels of each vaccine attribute (listed in Table 1) in deciding whether or not to accept a particular hypothetical TBE vaccine profile. Participants were shown three hypothetical profiles consisting of several attributes with varying levels on their screen and asked to select the most favorable profile of the three. Each participant was asked to do this 12 times, with different combinations of attribute levels shown each time (see Table 2 within Supplementary File 1). Equal efficacy and tolerability were assumed across all hypothetical TBE vaccine profiles; therefore, the discrete-choice analysis did not assess the importance of efficacy, side effect frequency, or side effect severity as attributes.

**Table 1:** Summary of TBE vaccine attributes and levels assessed in the discrete-choice experiments

	**Attribute**	**Attribute Label**	**Level 1**	**Level 2**	**Level 3**
1	Schedule	Vaccine dosing schedule	Dose 1: Day 0 Dose 2: Day 7 Dose 3: Day 21	Dose 1: Day 0 Dose 2: 1–3 mths Dose 3: 9–12 mths	Dose 1: Day 0 Dose 2: Day 14 Dose 3: 9–12 mths
2	Booster	Booster time interval	3 years	5 years	10 years
3	Switch	Ability to switch between different TBE vaccine brands	Yes	No	-
4	Cross-protection	Protection against different TBE strains	Yes	No	-
5	Origin	Vaccine manufacturer country of origin	Europe	USA	-
6	Packaging	Environmentally friendly packaging (e.g., fully plastic-free)	Yes	No	-
7	Access	Where to access the vaccine	Family doctor	Family doctor and somewhere other than family doctor, e.g., specialist vaccination center/ travel clinic/ pharmacist	-

Participants were asked to select a preferred TBE vaccine profile from a choice of three hypothetical profiles on their screen, repeating this process 12 times. Each profile was comprised of varying levels of each of the different attributes listed above to create distinct vaccine profiles to select from

*TBE* Tick-borne encephalitis

We used Sawtooth Software © (USA) to calculate utility scores for aggregated discrete choice data via regression modeling. Effects coding was applied to scale scores for each level within an attribute to equal zero. Therefore, utility scores should only be compared within, and not across different attributes, since scores are relative within each attribute. The higher the utility score, the greater perceived preference towards that attribute level, compared to the other levels for the same attribute. Conversely, the lowest utility score indicates that an attribute level is the least preferred option in the context of the other levels for that attribute.

Thereafter, weighted importance was calculated to determine how important each overall attribute was in influencing the selection of preferred hypothetical TBE vaccine profiles. Briefly, the range (max–min) of the average utility score across each attribute level was first determined. Attribute importance (weight) was then calculated as the range for each attribute divided by the sum of all attribute ranges to give percentage values, with higher values indicating greater overall importance of that attribute in influencing choice. Utility scores (standard error [SE]) and weighted importance were presented for the overall survey group.

### Statistical analysis

For the general quantitative survey, mean data were reported for the overall survey group, and according to age or gender. For comparisons between subgroup data, unpaired t-tests were used on mean percentages, comparing across two individual categories at a time.

Data from the discrete-choice questions are presented for the overall survey group and by endemic region, age, TBE vaccination status, children < 18 years old, or traveler versus resident of endemic area.

## Results

### Study disposition and baseline demographics

Initially, 4255 participants were screened. Nine hundred and ninety-three participants failed screening, with the most common reasons being answering ‘no’ to whether they traveled in endemic regions in Germany (*n* = 369/993), and ‘no’ to whether or not they were open to receiving vaccines (*n* = 290/993). Once the maximum demographics quotas were fulfilled for each country, a further 1883 participants were screened out and omitted from the survey.

In total, 1379 participants completed the survey: 500 from Germany (36.3%), 300 from Sweden (21.8%), 301 from Switzerland (21.8%), and 278 from Austria (20.2%). There was an almost even split in the proportion of male (52% [*n* = 710/1379]) and female (48% [*n* = 667/1379]) participants overall, and 84% were over 30 years old (*n* = 1161/1379) (Table 2). More than half of participants were in full or part-time employment (54.7% [*n* = 754/1379]). The majority of participants did not have children under the age of 18 years old (78.9% [*n* = 1088/1379]). Twenty-five percent (*n* = 342/1379) were vaccinated against TBE in the overall survey group. In Austria, 44% of participants (*n* = 121/278) were vaccinated (Table 2), though this was due to the fact that the 20% maximum vaccination quota had to be raised for Austria in order to meet the other minimum demographics quotas. A greater proportion of participants in the 18–30 (34% [*n* = 74/218]) and 31–50 (31% [*n* = 126/410]) age groups were vaccinated, compared with those in the 51–65 (19% [*n* = 77/400]) and > 65 (19% [*n* = 65/351]) age groups (*P* ≤ 0.05).

**Table 2:** Frequency and percentage distribution of selected demographic characteristics of participants

	**Germany** **(*n* = 500)**	**Sweden** **(*n* = 300)**	**Switzerland** **(*n* = 301)**	**Austria** **(*n* = 278)**	**Overall** **(*n* = 1379)**
**Age, years; n (%)**					
18–30	86 (17.2)	51 (17.0)	40 (13.3)	41 (14.7)	218 (15.8)
31–50	141 (28.2)	84 (28.0)	97 (32.2)	88 (31.7)	410 (29.7)
51–65	141 (28.2)	80 (26.7)	85 (28.2)	94 (33.8)	400 (29.0)
> 65	132 (26.4)	85 (28.3)	79 (26.2)	55 (19.8)	351 (25.5)
**Gender; n (%)**					
Male	261 (52.2)	149 (49.7)	159 (52.8)	141 (50.7)	710 (51.5)
Female	239 (47.8)	151 (50.3)	141 (46.8)	136 (48.9)	667 (48.4)
Other	0 (0)	0 (0)	1 (0.33)	1 (0.36)	2 (0.15)
Prefer not to say	0 (0)	0 (0)	0 (0)	0 (0)	0 (0)
**Employment status; n (%)**					
Full-/part-time employment	274 (54.8)	156 (52.0)	166 (55.1)	158 (56.8)	754 (54.7)
Unemployed	25 (5.0)	19 (6.3)	16 (5.3)	10 (3.6)	70 (5.1)
Retired	159 (31.8)	101 (33.7)	91 (30.2)	95 (34.2)	446 (32.3)
Student	29 (5.8)	12 (4.0)	12 (4.0)	11 (3.96)	64 (3.8)
Other	13 (2.6)	12 (4.0)	16 (5.3)	4 (1.44)	45 (3.3)
**Family status, n (%)**^**a**^					
Children < 18 years old	99 (19.8)	79 (26.3)	61 (20.3)	52 (18.7)	291 (21.1)
No children < 18 years old	401 (80.2)	221 (73.7)	240 (79.7)	226 (81.3)	1088 (78.9)
**TBE vaccination status; n (%)**^b^					
Vaccinated	101 (20.2)	60 (20)	60 (19.9)	121 (43.5)	342 (24.8)
Unvaccinated	399 (79.8)	240 (80)	241 (80)	157 (56.5)	1037 (75.2)

There was a minimum requirement to have 15% of participants in each age bracket (18–30, 31–50, 51–65, > 65 years old), a maximum of 65% for male or female gender category (other genders were not subject to minimum or maximum quotas), a maximum of 20% vaccinated against TBE, a minimum of 20% with children < 18 years old, and representation in all employment categories. Once each quota was met, any further participants within that demographic did not progress to the survey

*TBE* Tick-borne encephalitis

^a^Participants were asked if they had children under 18 years old living with them, for whom they made decisions for regarding vaccines

^b^Maximum vaccination quotas were raised for Austria in order to meet other demographic quotas

On the whole, baseline demographics were relatively similar across the different geographical locations. However, there were fewer male participants than female participants aged 18–30 years old overall (9.6% [*n* = 68/710] versus 22.3% [*n* = 149/667]), and more males than females over 65 years old (32.3% [*n* = 229/710] versus 18.3% [*n* = 122/667]). The proportion of Austrian participants over the age of 65 years old was also slightly lower compared with the overall survey group (20% [*n* = 55/278] versus 26% [*n* = 351/1379]), and individual endemic regions. In Sweden, the proportion of participants with dependent children under 18 years old was greater compared with the overall survey group (26.3% [*n* = 79/300] versus 21.1% [*n* = 291/1379]), or other endemic regions (Table 2).

Almost half of German participants traveled to, but did not live in, TBE-endemic regions in Germany (45% [*n* = 226/500]). The most commonly visited TBE-endemic regions were Bayern (72% [*n* = 162/226]), Baden-Württemberg (33% [*n* = 75/226]), and Thüringen (26% [*n* = 59/226]). Conversely, only 23% (*n* = 70/300) of Swedish participants traveled to, but did not live in, TBE-endemic regions in Sweden; namely Stockholm (54% [*n* = 38/70]), Blekinge (30% [*n* = 21/70]), and Kalmar (24% [*n* = 17/70]). Among the 24 out of 301 Swiss participants (8%) who did not live in TBE-endemic regions in Switzerland, the top three TBE-endemic regions for travel were Vaud (54% [*n* = 13/24]), Valais (50% [*n* = 12/24], and Bern (33% [*n* = 8/24]). These data are not reported for Austrian participants, given that TBE is considered to be focally endemic across all regions of Austria [4]. 

### TBE awareness, general vaccine knowledge and vaccine motivating factors

Approximately 79% of participants (*n* = 1085/1379) were aware of TBE. The proportion of participants who were unaware of TBE was greater among the 18–30 age group (34% [*n* = 74/218]), compared with the 31–50 (22% [*n* = 92/410]), the 51–65 (14% [*n* = 56/400]), and the over 65 age groups (21% [*n* = 72/351]) (*P* ≤ 0.05 for each comparison).

Overall, 60% of participants (*n* = 827/1379) claimed to have at least moderate knowledge of vaccines in general or higher (Likert scale 1–7; 1 = not at all knowledgeable; 7 = very knowledgeable); 26% (*n* = 362/1379) considered themselves to be knowledgeable or very knowledgeable (Supplementary Fig. 1). A numerically higher proportion of participants over the age of 65 years old considered themselves to be knowledgeable or very knowledgeable on vaccines (30% [*n* = 104/351]), compared with the overall survey group (26% [*n* = 413/1379]). This difference was greater still when comparing the over 65 age group with the 51–60 age group (30% [*n* = 104/351] versus 22% [*n* = 89/400]) (*P* ≤ 0.05; Supplementary Fig. 1).

**Fig. 1:**
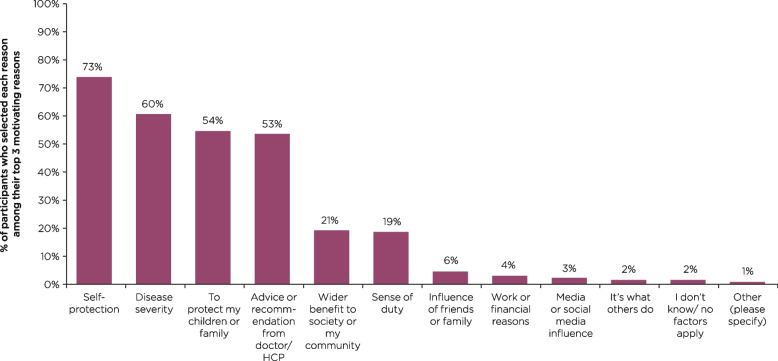
Motivating reasons for getting vaccinated HCP, healthcare professional. Participants were asked: *What are the most motivating reasons for getting yourself or your children vaccinated against infectious diseases? Please select your top three most motivating reasons.* Data are shown as the percentage of participants who selected each attribute among their top three motivating reasons for getting vaccinated among the overall survey group

For 73% of participants, self-protection was among their top three reasons to get vaccinated against any disease (*n* = 1003/1379; Fig. 1). Disease severity, protection of children or family, and doctor/HCP advice or recommendation were among the top three motivators for getting vaccinated for more than half of participants in the overall survey population (Fig. 1).

A summary of the most commonly selected reasons as being among the top three motivators for getting vaccinated against any disease are presented by age and gender subgroups in Supplementary Fig. 2. Briefly, self-protection was more important among the 51–65 (79% [*n* = 314/400]) and over 65 age groups (78% [*n* = 274/351]), compared with the 18–30 (68% [*n* = 149/218]) and 31–50 (65% [*n* = 266/410]) age groups (*P* ≤ 0.05 for each comparison). A similar pattern of findings was observed for advice or recommendation from a doctor/HCP. Disease severity was more important to participants aged 51–65 years old (63% [*n* = 253/400]), versus those aged 18–30 years old (55% [*n* = 120/218]) (*P* ≤ 0.05; Supplementary Fig. 2).

When comparing gender subgroups, a greater proportion of females (63% [*n* = 419/667]) than males (56% [*n* = 400/710]) (*P* ≤ 0.05) selected disease severity among their top three motivating reasons for getting vaccinated. Self-protection, on the other hand, was more important among males (76% [*n* = 537/710]) than females (70% [*n* = 465/667]) (*P* ≤ 0.05; Supplementary Fig. 2).

### Reliance on doctor/HCP for general vaccine knowledge

**Fig. 2:**
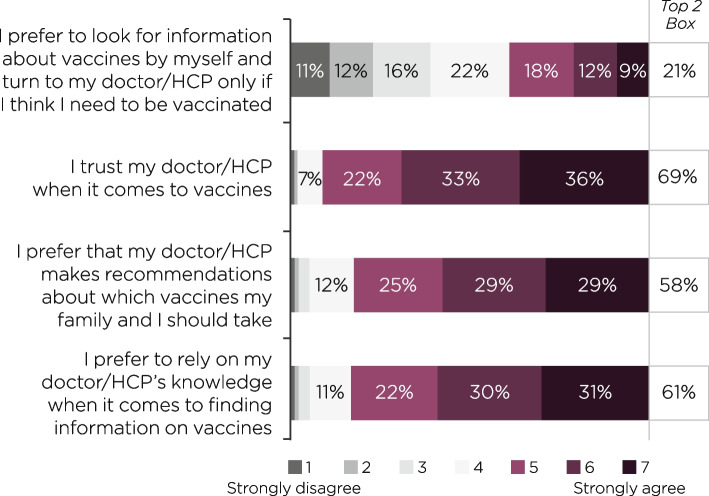
Doctor/HCP reliance for the overall study population HCP, healthcare professional. Participants were asked: *Please tell us whether you agree or disagree with each of the statements below on a Likert scale of 1–7, where 1* = *strongly disagree and 7* = *strongly agree.* ‘Top 2 Box’ values show the total % of participants who answered either agree or strongly agree (score of 6 or 7)

Participants were asked to state their agreement with four different statements relating to their reliance on their doctor/HCP for general vaccine advice and information, using a Likert-scaled response (1–7; 1 = strongly disagree; 7 = strongly agree). More than half (58–69%) of all participants agreed or strongly agreed that they trust their doctor/HCP when it comes to the subject of vaccines, that they rely on their doctor/HCP’s knowledge when it comes to finding information on vaccines, and that they prefer their doctor/HCP to make recommendations on which vaccines they or their families should take (Fig. 2).

A greater proportion of participants in the 51–65 (67% [*n* = 267/400]) and over 65 age groups (68% [*n* = 237/351]) strongly agreed that they prefer to rely on their doctor/HCP’s knowledge, than in the 18–30 (54% [*n* = 118/218]) and 31–50 (54% [*n* = 223/410]) age groups (*P* ≤ 0.05 for each comparison). Males were also more likely to rely on their doctor/HCP’s knowledge (66% [*n* = 471/710]), than females were (56% [*n* = 372/667]) (*P* ≤ 0.05).

Conversely, just 21% (*n* = 284/1379) of participants agreed or strongly agreed that they prefer to self-educate on vaccines, only turning to doctors/HCPs if they feel they need to be vaccinated (Fig. 2). A smaller proportion of participants in the over 65 years age group preferred to self-educate on vaccines (15% [*n* = 54/351]), compared with participants in the 18–30 (25% [*n* = 54/218]), 31–50 (22% [*n* = 91/410]), and 51–65 (21% [*n* = 85/400]) age groups (*P* ≤ 0.05 for each comparison).

### Attribute preferences for TBE vaccines

**Fig. 3:**
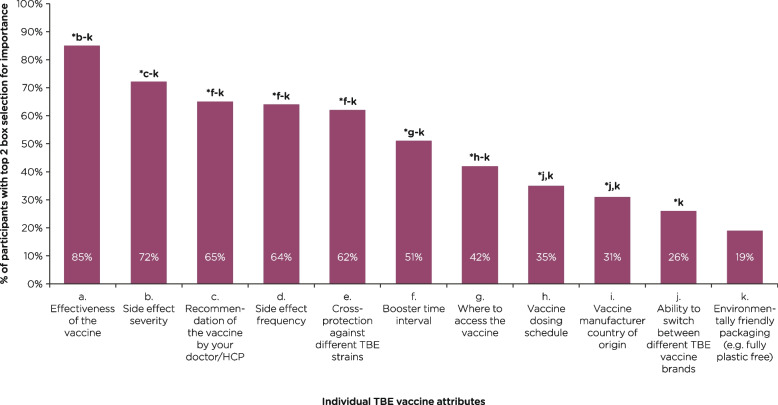
Stated importance of individual TBE vaccine attributes *Denotes *P*-value ≤ 0.05. HCP, healthcare professional; TBE, tick-borne encephalitis. Participants were asked: *Please rate the importance of each of these individual TBE vaccine characteristics on a scale of 1–7, where 1* = *not at all important and 7* = *extremely important.* Data are shown as the total % of participants who selected each attribute among the top 2 boxes for importance (i.e., 6, important; or 7, extremely important)

When asked to consider the stated importance of individual TBE vaccine attributes as standalone attributes (Likert-scale 1–7; 1 = not at all important and 7 = extremely important), a greater proportion of participants rated effectiveness of the vaccine (85% [*n* = 1169/1379]) and side effect severity (72% [*n* = 994/1379]) as important or extremely important, compared with all other attributes (*P* ≤ 0.05 for each comparison; Fig. 3). Side effect frequency (64% [*n* = 883/1379]), cross-protection against TBE strains from other endemic regions (62% [*n* = 854/1379), and booster interval (51% [*n* = 702/1379]) were rated important or extremely important for more than half of participants (Fig. 3). Environmentally friendly packaging appeared to be of least importance, with only 19% of participants (*n* = 261/1379) rating it as either important or extremely important as a standalone TBE vaccine attribute.

**Fig. 4:**
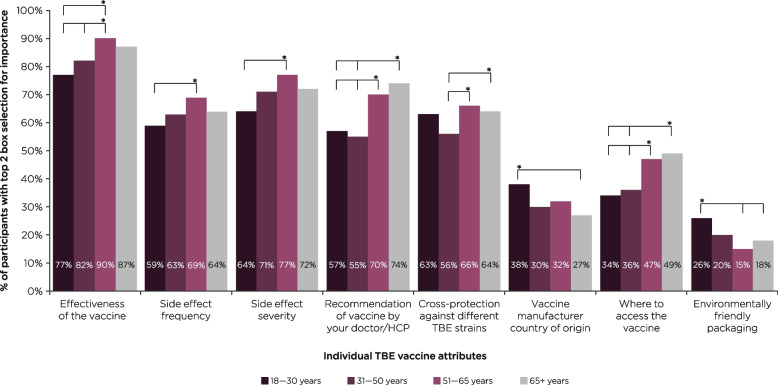
Summary of key differences in stated importance of individual TBE vaccine attributes according to age *Denotes *P*-value ≤ 0.05. HCP, healthcare professional; TBE, tick-borne encephalitis. Participants were asked: *Please rate the importance of each of these individual TBE vaccine characteristics on a scale of 1–7, where 1* = *not at all important and 7* = *extremely important.* Data are shown as the total % of participants who selected each attribute among the top 2 boxes for importance (i.e., important, or very important) among each age category

Key differences in the stated preferences for standalone TBE vaccine attributes between age subgroups are summarized in Fig. 4. When analyzing the proportion of participants who rated each attribute as important or very important, vaccine effectiveness was more important to participants aged 51–65 years old (90% [*n* = 359/400]), compared with participants aged 18–30 (77% [*n* = 167/218]) or 31–50 years old (82% [*n* = 338/410]) (*P* ≤ 0.05 for each comparison). Similarly, this attribute was more important among participants who were over 65 years old (87% [*n* = 305/351]), versus those who were aged 18–30 (77% [*n* = 167/218]) (*P* ≤ 0.05). Side effect frequency and severity were both more important to participants aged 51–65 years old, compared with those aged 18–30 (frequency: 69% [*n* = 276/400] versus 59% [*n* = 128/218]; severity: 77% [*n* = 309/400]) versus 64% [*n* = 140/218]) (*P* ≤ 0.05 for each comparison; Fig. 4). Furthermore, side effect frequency was also more important among females (69% [*n* = 458/667]), than males (60% [*n* = 429/710]) (*P* ≤ 0.05). Cross-protection against different TBE strains was more important to participants aged 51–65 years old (66% [*n* = 263/400]) and those over 65 years old (64% [*n* = 223/351]), compared with participants in the 31–50 age group (56% [*n* = 231/410]) (*P* ≤ 0.05 for each comparison; Fig. 4).

There were no notable differences between age groups in the stated importance of booster time interval, dosing schedule, or ability to switch TBE vaccine brands; nor were there any differences in the stated importance of TBE vaccine attributes between males and females, other than side effect frequency.

**Fig. 5:**
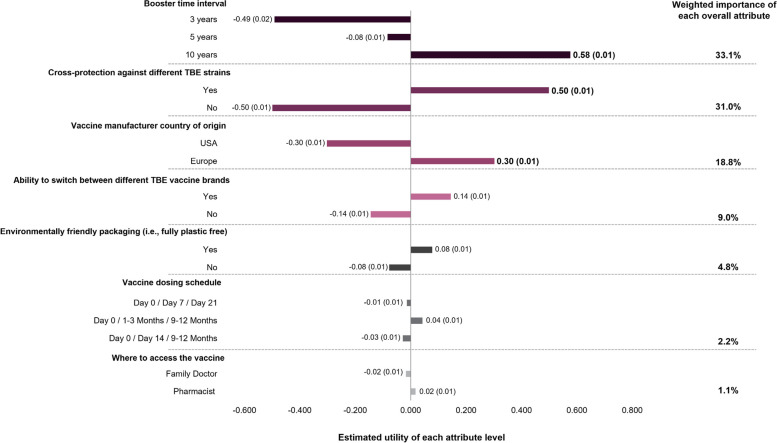
Preference for individual attribute levels (utility) and overall importance of each attribute (weighted importance) in the selection of hypothetical TBE vaccine profiles TBE, tick-borne encephalitis. Conjoint analysis was conducted on data from discrete-choice questions to determine the perceived importance (utility) of each attribute level when selecting a preferred hypothetical TBE vaccine profile. Higher utility scores indicate greater preference towards that attribute level, in the context of other levels for the same attribute, in selecting a preferred hypothetical TBE vaccine profile. Comparisons can only be made between levels within each attribute, and not between different attributes. Utility data are shown as: utility score (standard error); scores for each level within an attribute are normalized to sum to 0. The weighted importance of each overall attribute on influencing the selection of a preferred TBE vaccine profile was determined by calculating the range (max–min) of the average utility score across each attribute level. Attribute importance (weight) was then calculated as the range for each attribute divided by the sum of all attribute ranges. Weighted importance data are presented as percentage values, with higher values indicating greater importance of that attribute in influencing choice

In the discrete-choice questions, booster time interval and cross-protection against other geographically distinct TBE strains were the most important attributes in influencing the selection of a preferred hypothetical TBE vaccine profile (weighted importance: 33.1% and 31.0%, respectively; Fig. 5). Conversely, vaccine dosing schedule and where to access the vaccine appeared to be the least important attributes influencing vaccine decision-making (weighted importance: 2.2% and 1.2%, respectively; Fig. 5). These findings were consistent across endemic regions, and across subgroups based on age, gender, employment status, and parental status (Supplementary Table 1).

When examining the importance of individual attribute levels on influencing the selection of a preferred hypothetical TBE vaccine profile, the strongest indication of preference was towards a 10-year booster interval (utility score: 0.58 [SE: 0.01]), rather than 3- or 5-year intervals (utility score: -0.49 [SE:0.02] and -0.08 [SE:0.01], respectively). This was closely followed by the option to gain cross-protection against other TBE strains (utility score: 0.50 [SE: 0.01]) versus no cross-protection (utility score: -0.50 [SD: 0.01]; Fig. 5). There were no strong preferences towards any of the individual attribute levels for vaccine dosing schedule or where to access the vaccine, consistent with weighted importance data, demonstrating the lack of influence these attributes played in hypothetical TBE vaccine selection (Fig. 5).

## Discussion

Findings from this real-world survey among people living in four TBE-endemic regions in Europe demonstrated a high level of self-declared general vaccine knowledge and a high awareness of TBE among the overall survey group, which was highly consistent with previous European studies [24]. In accordance with previous reports of general vaccine uptake, the majority of participants trusted and relied upon their doctor/HCP for vaccine recommendations, highlighting the pivotal role doctors/HCPs play in supporting patients in their vaccine decision-making [28].

Self-protection was the most motivating factor for getting vaccinated, though disease severity, protection of children or family, and advice or recommendation from their doctor/HCP also rated highly for over half of participants. As anticipated, most participants rated vaccine effectiveness and side effect frequency as the most important standalone TBE vaccine attributes to consider, though more than half also ranked booster time interval and cross-protection against other geographically distinct TBE strains as important or very important.

The importance of booster time interval and cross-protection was further validated by the results of the discrete-choice analysis, with these attributes ranking most influential in the context of selecting a preferred hypothetical TBE vaccine. The 10-year booster interval was the most desirable attribute level, compared with 3- or 5-year intervals, when participants were asked to choose a preferred hypothetical TBE vaccine profile; while profiles offering cross protection against other TBE strains were greatly preferred, versus those with no cross-protection.

The desire for cross-protection may not be surprising, given the current spread of all three variants of TBEV in the Baltic States, and the European and Siberian variants in Finland [2, 13, 14]. However, the reasons for the preference towards a longer booster interval are less clear. It could be speculated that a mixture of the convenience of an extended booster schedule and the perception of enhanced potency of a vaccine with a longer booster interval may have influenced decision-making in this study. It should be noted, however, that discrete-choice findings demonstrate preferences within the context of other available choices; therefore, if the attribute levels were extended to a longer or shorter booster interval, then a new level could conceivably out-rank the 10-year booster in terms of preference.

Nonetheless, in the context of this survey, there was a clear preference for a longer booster interval, compared with 3- or 5-year intervals [18, 19], which could perhaps explain why compliance with current booster recommendations of once every 3–5 years is consistently low across Europe [24]. It is conceivable that recommendations for longer booster intervals could improve vaccine compliance, and thus help to lower the incidence of TBE cases in Europe. Indeed, the recommending body in Switzerland has already updated its recommendations for TBE vaccine boosters from once every three years to once every 10 years [29]. Promisingly, findings from a retrospective analysis conducted in Switzerland between 2006–2020 suggest that TBE vaccine effectiveness did not decrease among fully vaccinated individuals even when their last dose was 10 or more years ago [30]. Further, TBE surveillance data collected during this period indicate that there was no evidence of increased breakthrough TBE associated with extended booster intervals among any age group and that acceptability of the TBE vaccine was markedly improved among the Swiss population [31]. Alongside existing and future effectiveness data, our observation of a clear preference towards this kind of extended booster interval could be used to guide updates to current prescribing information for existing TBE vaccines and to inform European-wide updates to TBE vaccine recommendations in line with country-specific risk, which may help to improve vaccine uptake.

One surprising observation in the current study was that dosing schedule was less important in TBE vaccine decision-making among our participants, with no clear preference towards any of the dosing schedules presented. Rather than suggesting that dosing schedule is not important to vaccinees, it may be that none of the levels presented within this survey were particularly desirable among our participants. For individuals identified as high-risk or those in need of immediate protection, Encepur has an approved rapid dosing schedule, whereby all three doses can be given over a 21-day period, with a first booster 12–18 months later [18]. The lack of preference towards this rapid dosing schedule in the current study could perhaps indicate a lack of perceived TBE risk among our survey participants, translating to a lack of urgency in seeking immediate and full protection, though this would require further exploration.

Additionally, although manufacturer country of origin was within the top three most important attributes for influencing decision-making in the discrete-choice analysis, its weighted importance was still markedly lower than that of booster interval or cross-protection, suggesting that it was of lower importance than the aforementioned attributes in hypothetical TBE vaccine decision-making. As a stand-alone attribute, only a third of participants regarded it as highly important, suggesting that while it may be a consideration for some, the majority of participants may not necessarily feel a strong emotional preference towards a vaccine based solely on where it is manufactured.

We observed some interesting differences between demographic subgroups in terms of knowledge, doctor/HCP reliance, vaccine motivation, and preferences for specific TBE vaccine attributes. For example, we noted a greater emphasis on self-protection among older participants, coupled with greater self-proclaimed vaccine knowledge. Despite this, a greater proportion of older participants relied on doctors/HCPs for vaccine advice and recommendations, compared with younger participants. Furthermore, older participants placed a greater importance on disease severity as a motivating factor for getting vaccinated, suggesting a heightened awareness or concern for health with increasing age, which is not unexpected. Nonetheless, fewer participants over the age of 50 years old were vaccinated, suggesting a need to improve uptake specifically within this age group. We noted a similar pattern of findings based on employment status, in that key differences existed mainly between retired participants versus students, suggesting that age was more likely the determining factor. With that in mind, and in the interest of brevity, we did not report data by employment status, other than for the discretechoice experiment.

Interestingly, when comparing between genders, self-protection was more important as a motivating factor for getting vaccinated among males than females. Notably, the proportion of participants over the age of 65 years was greater among males than females, suggesting that this observation may have been at least partly age-related, rather than gender-related. Additionally, we did not observe a greater emphasis on protecting family among females to counter this observation, as might have been expected.

Although we do not report data by endemic region with the exception of discrete-choice data, we did note some differences in vaccine motivation among Swedish participants, compared with participants from other endemic regions, which may be explored in a future publication. For example, while disease severity was a more motivating factor in Sweden, advice from a doctor/HCP and sense of duty were less motivating reasons for getting vaccinated among this group, compared with participants from other regions (data not shown). This could be attributable to the higher proportion of Swedish participants who declared themselves to be knowledgeable/ very knowledgeable on TBE, suggesting a level of self-reliance among these participants, though further exploration is required to validate this observation.

As stand-alone vaccine attributes, effectiveness, side effect frequency and severity, doctor/HCP recommendation, cross-protection, and where to access the vaccine were rated more important among older versus younger participants, while side effect frequency was more important to females than males. We did not observe any notable differences in the stated importance of booster interval between subgroups. Furthermore, in the context of selecting a preferred hypothetical TBE vaccine profile, there were no substantial differences in the importance of each attribute, nor any differences in preferences towards specific attribute levels, between demographic subgroups. These findings suggest that our overall observation of a greater preference towards a 10-year booster interval, compared to 3- or 5-yearly boosters, along with the desirability of cross-protection against other TBE strains, could be extended to a wider demographic, irrespective of age, gender, or nationality.

Novel insights, such as those we’ve shared here, on how TBE awareness, doctor/HCP reliance, and vaccine motivation can vary on an individual basis, and perhaps from country to country, could inform doctors/HCPs on how to offer a more personalized approach when recommending or prescribing TBE vaccines. They may also form the basis of more targeted public health education campaigns for at-risk groups or specific demographics, such as younger vaccinees whose general vaccine or TBE knowledge may be lacking. Our findings also highlight a unique opportunity to educate doctors/HCPs and patients on the efficacy, safety, and cross-reactivity of current TBE vaccines, given the importance of these attributes across the board. This approach may help to alleviate existing fears or anxieties around TBE vaccination or to clear up any misconceptions about existing vaccines, in the hope of improving TBE vaccine uptake.

There are some potential limitations when conducting a survey such as this, in that market research panels can be inherently biased. By introducing demographic quotas, we tried to ensure that our survey population was as representative of the general adult population in each of the areas surveyed as possible. Having relatively low maximum quotas on TBE vaccination rates for each region enabled us to specifically recruit a large number of unvaccinated individuals, whose novel insights may help to elucidate barriers to TBE vaccination among unvaccinated individuals in TBE-endemic countries. However, as this survey was conducted across four independent countries, some caution should be exercised before generalizing results across Europe. Notably, we observed fewer differences in responses between country-to-country subgroups than with age or gender comparisons (data not shown), suggesting that, for the most-part, nationality did not influence opinion within this study to the same extent as age or gender. Nonetheless, a larger future survey across more TBE-endemic regions, with a focus on country-to-country comparisons, would provide further insight. It also highly likely that there are other factors which may influence preferences and attitudes towards vaccination, which were not covered in this survey, such as individual health status, ethnicity, and religious beliefs, in addition to various social and contextual factors. Future studies may also wish to explore some of these to further our understanding.

Comprehension can also be an issue with this type of study, therefore a number of pre-tests were carried out to ensure that language was clear and concise throughout, and that participants clearly understood each part of the survey.

## Conclusions

Self-protection, disease severity, doctor/HCP recommendation, and protection of children or family were the top reasons for seeking any vaccine among participants of this survey. Most people trusted their doctor/HCP’s advice when it comes to vaccinations, and many rely upon it. When making decisions on whether or not to receive a TBE vaccine, efficacy and safety were the most important standalone vaccine attributes to consider. When equal efficacy and safety were assumed, hypothetical TBE vaccine profiles with a 10-year booster interval were selected most often compared with profiles with a 3- or 5- year interval, indicating the desirability of this attribute level among participants. Cross-protection against other geographically distinct TBE strains was also highly desirable, compared with vaccine profiles with no cross-protection. Clear differences existed between age and gender subgroups in terms of vaccine knowledge, vaccine motivations, and preferred TBE vaccine attributes. These observations highlight the unique opportunity for doctors/HCPs to better understand the individual needs of each patient for a more personalized approach to shared vaccine decision-making, and the need for more targeted public health campaigns. Understanding motivators and preferences around vaccination among people living in TBE-endemic countries in Europe is essential for helping to reduce the incidence of TBE by increasing vaccine uptake, continuing to update TBE vaccine labels based on new data, and continually monitoring and updating vaccine recommendations in accordance with changing risk.

## Supplementary Information

Supplementary Material 1.

Supplementary Material 2.

## Data Availability

Any request for data are welcome to be directed to the corresponding author with a justification for the request.

## Abbreviations

EMPHRA: European Pharmaceutical Market Research Association
ESOMAR: European Society for Opinion and Market Research
HCP: Healthcare professional
TBE: Tick-borne encephalitis
TBEV: Tick-borne encephalitis virus
