# Supplementary material for: What makes patients tick? Vaccine preferences against tick-borne encephalitis in four European countries
Source: BMC Infect Dis. 2024 Oct 13;24:1151. doi: 10.1186/s12879-024-10045-4 (PMC11472448; doi:10.1186/s12879-024-10045-4)
Supplement: Supplementary file 1 — Supplementary Material 1. [file 12879_2024_10045_MOESM1_ESM.pdf]

TBE Vaccine Patient Preference

Questionnaire

V8_21st August 2023

Note: purple **text represents command information for the survey programer. Participants of the survey are only shown text in** blue**.**

SCREENER

S1. Country

Base for question	All
Programming	Single code
Question text	N/a
Respondent instruction	
Codes	
01	Germany
03	Sweden
04	Switzerland
05	Austria

S1a. Language preference

Base for question	Switzerland (S1_04)
Programming	Single code
Question text	Which language would you like to complete the questionnaire in?
Respondent instruction	
Codes	
02	French
03	German

I2 Screening intro

Base for question	All
Programming	Information screen.
Question text	Thank you. You will now be asked a maximum of 7 questions to check that you are suitable to participate in this survey.
Respondent instruction	

S2. Age

Base for question	All	
Programming	Single code	
Question text	Which age category do you fall under?	
Respondent instruction		
01	younger than 18 years old	THANK AND CLOSE
02	18-30 years old	CHECK QUOTAS
03	31-50 years old	
04	51-65 years old	
05	older than 65 years old	

S3. Gender

Base for question	All

Programming	Single code	
Question text	What gender do you identify as?	
Respondent instruction		
01	Male	CHECK QUOTAS
02	Female	
03	Other	
04	Prefer not to say	

S4a. German State

Base for question	Germany only	
Programming	Single code	
Question text	Which state do you primarily live in?	
Respondent instruction		
01	Baden-Württemberg	CONTINUE TO S6
02	Bayern	CONTINUE TO S6
03	Berlin	CONTINUE TO S4b
04	Brandenburg	CONTINUE TO S4b
05	Bremen	CONTINUE TO S4b
06	Hamburg	CONTINUE TO S4b
07	Hessen	CONTINUE TO S6
08	Mecklenburg-Vorpommern	CONTINUE TO S4b
09	Niedersachsen	CONTINUE TO S4b
10	Nordrhein-Westfalen	CONTINUE TO S4b
11	Rheinland-Pfalz	CONTINUE TO S4b
12	Saarland	CONTINUE TO S4b
13	Sachsen	CONTINUE TO S6
14	Sachsen-Anhalt	CONTINUE TO S4b
15	Schleswig-Holstein	CONTINUE TO S4b
16	Thüringen	CONTINUE TO S6

S4b. German state travel

Base for question	Germany ONLY<br>All who do not select codes 01,02,07,13,16 at S4a	
Programming	Single code	
Question text	Do you ever travel to any of the below states for a vacation that involves outdoor activities, such as hiking, dog walking or camping?	
Respondent instruction		
01	Baden-Württemberg	CONTINUE TO S6
02	Bayern	
07	Hessen	
13	Sachsen	
16	Thüringen	
99	None of the above	CLOSE AT END OF SCREENER

S5a. Swedish state

**Base for question**	**SE only**	
**Programming**	**Single code**	
**Question text**	Which state do you primarily live in?	
**Respondent instruction**		
**01**	Stockholm	**CONTINUE TO S6**
**02**	Uppsala	**CONTINUE TO S6**
**03**	Södermanland	**CONTINUE TO S6**
**04**	Östergötland	**CONTINUE TO S6**
**05**	Jönköping	**CONTINUE TO S6**
**06**	Kronoberg	**CONTINUE TO S5b**
**07**	Kalmar	**CONTINUE TO S6**
**08**	Gotland	**CONTINUE TO S6**
**09**	Blekinge	**CONTINUE TO S6**
**10**	Skåne	**CONTINUE TO S5b**
**11**	Halland	**CONTINUE TO S5b**
**12**	Västra Götaland	**CONTINUE TO S6**
**13**	Värmland	**CONTINUE TO S6**
**14**	Örebro	**CONTINUE TO S5b**
**15**	Västmanland	**CONTINUE TO S6**
**16**	Dalarna	**CONTINUE TO S6**
**17**	Gävleborg	**CONTINUE TO S5b**
**18**	Västernorrland	**CONTINUE TO S5b**
**19**	Jämtland	**CONTINUE TO S6**
**20**	Västerbotten	**CONTINUE TO S5b**
**21**	Norrbotten	**CONTINUE TO S5b**

S5b. Swedish state travel

**Base for question**	**SE only**	
**Programming**	**Single code**	
**Question text**	Do you ever travel to any of the below states for a vacation that involves outdoor activities, such as hiking, dog walking or camping?	
**Respondent instruction**		
**01**	Stockholm	**CONTINUE TO S6**
**02**	Uppsala	
**03**	Södermanland	
**15**	Västmanland	
**04**	Östergötland	
**12**	Västra Götaland	
**08**	Gotland	
**16**	Dalarna	
**05**	Jönköping	
**19**	Jämtland	
**07**	Kalmar	

13	Värmland	
09	Blekinge	
99	None of the above	CLOSE AT END OF SCREENER

S5c. Switzerland state

Base for question	Switzerland only	
Programming	Single code	
Question text	Which state do you primarily live in?	
Respondent instruction		
01	Zurich	CONTINUE TO S6
02	Bern / Berne	CONTINUE TO S6
03	Luzern	CONTINUE TO S6
04	Uri	CONTINUE TO S6
05	Schwyz	CONTINUE TO S6
06	Unterwalden	CONTINUE TO S6
07	Glarus	CONTINUE TO S6
08	Zug	CONTINUE TO S6
09	Freiburg / Fribourg	CONTINUE TO S6
10	Solothurn	CONTINUE TO S6
11	Basel	CONTINUE TO S6
12	Schaffhausen	CONTINUE TO S6
13	Appenzell	CONTINUE TO S6
14	Sankt Gallen	CONTINUE TO S6
15	Graubünden	CONTINUE TO S6
16	Aargau	CONTINUE TO S6
17	Thurgau	CONTINUE TO S6
18	Ticino	CONTINUE TO S5d
19	Vaud	CONTINUE TO S6
20	Valais / Wallis	CONTINUE TO S6
21	Neuchâtel	CONTINUE TO S6
22	Genève	CONTINUE TO S5d
23	Jura	CONTINUE TO S6

S5d. Switzerland state travel

Base for question	Switzerland only	
Programming	Single code	
Question text	Do you ever travel to any of the below states for a vacation that involves outdoor activities, such as hiking, dog walking or camping?	
Respondent instruction		
01	Zurich	CONTINUE TO S6
02	Bern / Berne	
03	Luzern	
04	Uri	
05	Schwyz	
06	Unterwalden	

**07**	Glarus	
**08**	Zug	
**09**	Freiburg / Fribourg	
**10**	Solothurn	
**11**	Basel	
**12**	Schaffhausen	
**13**	Appenzell	
**14**	Sankt Gallen	
**15**	Graubünden	
**16**	Aargau	
**17**	Thurgau	
**19**	Vaud	
**20**	Valais / Wallis	
**21**	Neuchâtel	
**23**	Jura	
**99**	None of the above`	**CLOSE AT THE END OF THE SCREENER**

S6 Openness to vaccines

**Base for question**	**ALL**	
**Programming**	**Single code**	
**Question text**	Which of the following statements best describes your openness towards receiving vaccinations?<br><br>(A vaccination is defined as a product that offers protection from a disease. It is typically given as an injection.)	
**Respondent instruction**		
**01**	I **am** open to receiving vaccinations that help protect against infectious diseases	**CONTINUE**
**02**	I **am not** open to receiving vaccinations that help protect against infectious diseases	**CLOSE AT END OF SCREENER**
**03**	I don't know	
**04**	I prefer not to say	

S7 TBE vaccination status

**Base for question**	**ALL**
**Programming**	**Single code**
**Question text**	Tick-borne encephalitis (TBE) is a viral infection spread by tick bites. TBE is an infection of the central nervous system that affects the brain, spinal cord, and the linings around them. Ticks infected with the virus are found in various parts of Europe and Asia.<br><br>Have you ever personally received a vaccine to protect against a disease called Tick-borne encephalitis (TBE)?

**Respondent instruction**		
**01**	Yes	**CHECK QUOTA**
**02**	No	**CHECK QUOTA**

S8 Family status

**Base for question**	**ALL**	
**Programming**	**Single code**	
**Question text**	Do you have any children under the age of 18 years old living with you, who you make decisions for regarding vaccines or general healthcare?	
**Respondent instruction**		
**01**	Yes	**CHECK QUOTA**
**02**	No	**CHECK QUOTA**

S9 Employment status

**Base for question**	**All**	
**Programming**	**Single code. Randomise order of codes 1-4**	
**Question text**	Which of the below best describes your current employment status?	
**Respondent instruction**		
**01**	I am currently in full / part time employment	**Continue – aim for a mix**
**02**	I am unemployed	**Continue – aim for a mix**
**03**	I am retired	**Continue – aim for a mix**
**04**	I am a student	**Continue – aim for a mix**
**99**	Other	**Continue**

Main Questionnaire: Section A (3 mins)

I4. Section introduction

Base for section	All
Programming	Information screen
Question text	This section is about your views on vaccines generally.
Respondent instruction	

A1. General vaccine knowledge levels

Base for question	All
Programming	Scale. Single code
Question text	On a scale of 1-7, with 1 being not at all knowledgeable and 7 being very knowledgeable, how knowledgeable would you say are when it comes to **vaccines**?<br><br>(A vaccination is defined as a product that offers protection from a disease. It is typically given as an injection.)
Respondent instruction	
Rows	
01	1 – Not at all knowledgeable
02	2
03	3
04	4
05	5
06	6
07	7 – Very knowledgeable

A2 Vaccines reliance on HCP

Base for question	All
Programming	Rating scale (1-7). Randomise order of rows
Question text	We'd now like you to tell us your level of agreement with each of the below statements.
Respondent instruction	*Please tell us whether you agree or disagree with each of the below statements on a scale of 1-7 where 1=strongly disagree and 7=strongly agree*
Rows	
01	I prefer to rely on my doctor/healthcare professional's knowledge when it comes to finding information on vaccines
02	I prefer that my doctor/healthcare professional makes recommendations about which vaccines me and my family should take
03	I trust my doctor/healthcare professional when it comes to the subject of vaccines

04	I prefer to look for information about vaccines by myself and turn to healthcare professionals only if I think I need to be vaccinated
Columns	
01	Strongly disagree
02	
03	
04	
05	
06	
07	Strongly agree

A3. Vaccine motivating factors

Base for question	All
Programming	Top 3 multicode selection. Root codes 98 and 99 at the bottom (99 is a single code)
Question text	What are the most motivating reasons for getting yourself or your children vaccinated against infectious diseases?
Respondent instruction	*Please select the top 3 most motivating reasons*
Rows	
01	Wider benefit to society or my community
02	To protect my children or family
03	To protect myself
04	A sense of duty
05	It's what other people do
06	Advice or recommendation from a doctor or healthcare professional
07	The seriousness or severity of the disease or infection
08	The influence of friends or family
09	Work or financial reasons
10	Media or social media campaigns or influence
98	Other - please specify [OPEN TEXT BOX]
99	I don't know / no factors apply

Main Questionnaire: Section B (5 mins)

I5. Section introduction

Base for section	All
Programming	Information screen
Question text	This next section is about tick-borne encephalitis (TBE) vaccines
Respondent instruction	

B1. TBE awareness

Base for question	All	
Programming	Single code	
Question text	Were you aware of the disease called Tick-borne encephalitis (TBE) before taking this survey today?	
Respondent instruction		
01	Yes	
02	No	

I6. TBE and TBE vaccines info screen

Base for question	All
Programming	Information screen – no response needed<br><br>Allow for 20 seconds before allowing respondent to click 'next'
Question text	Please take the time to read the information below on Tick-borne encephalitis (TBE) and the vaccines that can be used to protect from it:<br><br>**TBE is a viral infection spread by tick bites. It is an infection of the central nervous system that affects the brain, spinal cord, and linings around them. Ticks infected with the TBE virus are found in various parts of Europe and Asia. They are usually found in rural or forest areas, but also in urban areas like city parks and gardens.**<br><br>**Vaccines build up your immunity (the body's ability to fight infection) to a particular disease. Several different vaccines are available to help prevent TBE infection and to protect against serious complications of the disease if a person does become infected. A TBE vaccine is given before any potential exposure to ticks or the disease itself. TBE vaccines are given as a series of injections.**<br><br>**TBE vaccines are available for children and adults of all ages.**

	To provide basic immunization (protection against TBE), 3 injections are needed at different timepoints.	
Respondent instruction		

I7. Vaccine attributes information screen

Base for question	All	
Programming	Information screen – no response needed.<br><br>Please allow for 30 seconds before allowing respondent to click 'next'	
Question text	We will now show you a list of TBE vaccine characteristics that people may consider before accepting / choosing a TBE vaccine for themselves or their family.<br><br>Different brands of TBE vaccine can vary in their characteristics.<br><br>Please take the time to read the text below carefully, including the explanation for each characteristic.	
Respondent instruction		
01	SHOW TABLE 1	

Table 1

TBE vaccine characteristic:	Explanation	Visualisation
**Vaccine dosing schedule**	To provide basic immunization (protection against TBE), 3 injections are needed at different timepoints.<br><br>The first injection is done at 'Day 0'. The final 2 injections can be spread across different time points, across weeks or months.<br><br>This is called the vaccine dosing schedule.	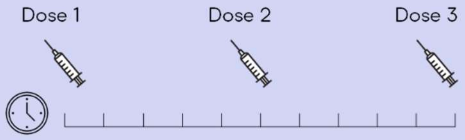
**Booster time interval**	To keep your immunity (protection) high, vaccinations need to be repeated.<br><br>These repeat vaccines are called boosters.<br><br>Booster vaccines can be given at various time intervals, often years after receiving the original vaccine.	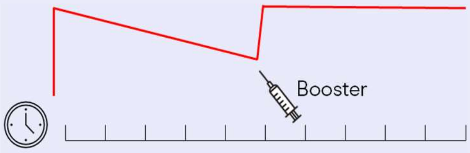

**Ability to switch between different TBE vaccine brands**	This means that you can swap between different TBE vaccine brands during your 3-dose vaccine schedule or have a different brand of vaccine as your booster vaccine and still be fully protected against TBE.	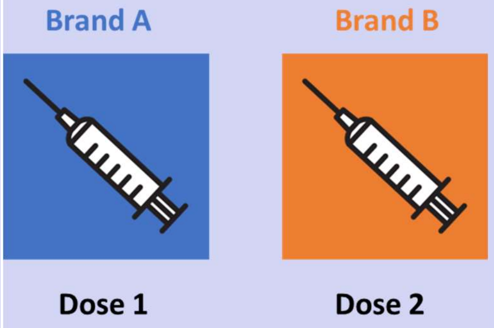
**Protection against TBE strains from separate geographical regions**	The TBE virus can have different “forms” called strains that can all make you sick.<br><br>There can be different strains in different parts of the world.<br><br>A vaccine can be protective against several of these strains.	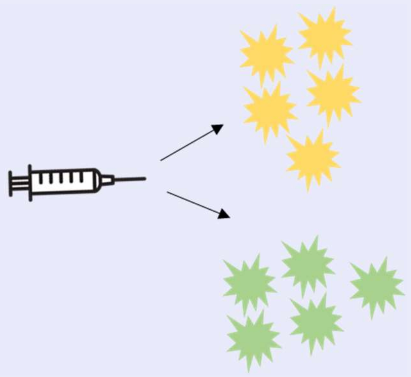
**Vaccine manufacturer country origin**	The companies that make each brand of TBE vaccine may have different countries/continents of origin.	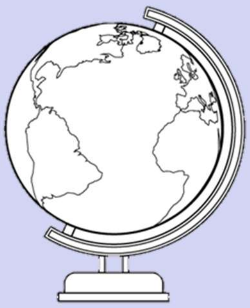

**Environmentally friendly packaging (e.g., fully plastic-free)**	TBE vaccines are delivered in already-filled syringes that are packed for protection and shipping. These could contain plastic or be plastic-free.	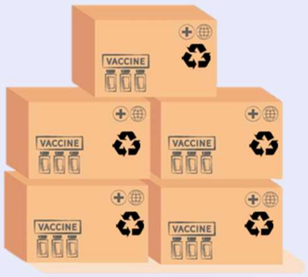
**Where to access the vaccine**	The place you or your family would need to go to receive the vaccine as an injection.	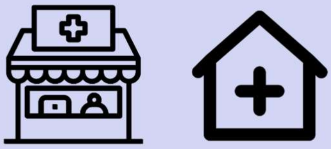
**Effectiveness of the vaccine**	The effectiveness of a vaccine means how much protection it gives you against the likelihood of contracting the disease.<br><br>Different TBE vaccines may have different levels of effectiveness.	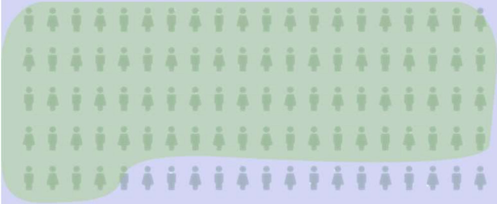
**Vaccine side-effect frequency**	An unwanted or unexpected effect of a medicine is called a side-effect, or an adverse event.<br><br>Each TBE vaccine brand may be associated with a different side-effect profile.<br><br>TBE vaccines can vary in terms of the frequency of their side effects – this is	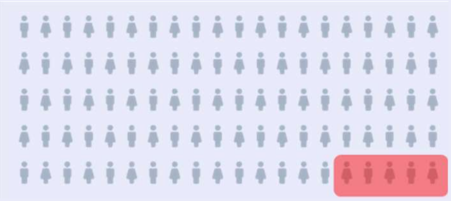

	how many people are likely to be affected by side-effects based on scientific studies.	
**Vaccine side effect severity**	TBE vaccines can vary in terms of the types of side-effects people might experience after having the vaccine.<br><br>Some side effects may be more serious or severe than others.	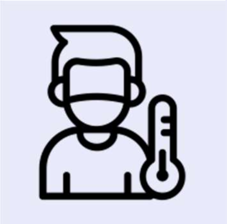
**Recommendation of the vaccine by your doctor or healthcare professional**	Whether the specific vaccine brand or product has been recommended to you by your doctor or healthcare professional.	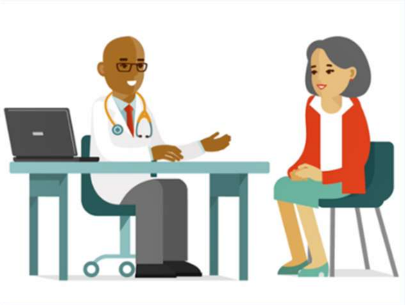

Main Questionnaire: Section C – Conjoint Analysis (~10 mins)

Conjoint exercise

SCREEN 1

In the next exercise, we'd like you to think about the choices you make or would make when deciding on which TBE vaccine to accept. This will help us understand the characteristics that would be important to you when choosing or accepting a TBE vaccine.

SCREEN 2

Please read the below carefully:

Following these instructions there will be 12 similar screens. On each screen, we will present you with 3 different imaginary TBE vaccine profiles.

Please select the TBE vaccine profile that would be your preferred choice for you or your family, from those shown.

For the purposes of this exercise, please assume that each vaccine profile has the same level of effectiveness and side effect severity and frequency. Please assume the effectiveness of each vaccine profile is high and the side effect frequency and severity is low.

Please note: although some of the individual characteristics appear several times, the combination of them will be different, so please read through each profile carefully.

SCREEN 3
C1. ALL RESPONDENTS

Please choose your preferred TBE vaccine profile

Please click on the characteristic name if you would like to see its explanation again

Conjoint screen mock-up		Vaccine 1	Vaccine 2	Vaccine 3	Vaccine 4
ATTRIBUTE 1	01	LEVEL INSERTED HERE	LEVEL INSERTED HERE	LEVEL INSERTED HERE	LEVEL INSERTED HERE
ATTRIBUTE 2	02	LEVEL INSERTED HERE	LEVEL INSERTED HERE	LEVEL INSERTED HERE	LEVEL INSERTED HERE
ATTRIBUTE 3	03	LEVEL INSERTED HERE	LEVEL INSERTED HERE	LEVEL INSERTED HERE	LEVEL INSERTED HERE
ATTRIBUTE 4	04	LEVEL INSERTED HERE	LEVEL INSERTED HERE	LEVEL INSERTED HERE	LEVEL INSERTED HERE
ATTRIBUTE 5	05	LEVEL INSERTED HERE	LEVEL INSERTED HERE	LEVEL INSERTED HERE	LEVEL INSERTED HERE

ATTRIBUTE 6	06	LEVEL INSERTED HERE	LEVEL INSERTED HERE	LEVEL INSERTED HERE	LEVEL INSERTED HERE
ATTRIBUTE 7	07	LEVEL INSERTED HERE	LEVEL INSERTED HERE	LEVEL INSERTED HERE	LEVEL INSERTED HERE
From the choices above, which option do you prefer?					
Preferred vaccine		01	02	03	04
C1b. In a hypothetical situation, would you be willing to accept a vaccine with the profile you selected to protect against TBE (for you or your family)?					
	01	Yes, I would accept / receive this vaccine			
	02	No, I would not accept / receive this vaccine			

CONJOINT DESIGN: EACH RESPONDENT TO BE SHOWN ~12 SCREENS

Table 2

Attribute list with levels		Level 1	Level 2	Level 3
Vaccine dosing schedule	01	Dose 1: Day 0<br>Dose 2: Day 7<br>Dose 3: Day 21	Dose 1: Day 0<br>Dose 2: 1-3 months<br>Dose 3: 9-12 months	Dose 1: Day 0<br>Dose 2: Day 14<br>Dose 3: 9-12 months
Booster time interval	02	3 years	5 years	10 years
Ability to switch between different TBE vaccine brands	03	Yes	No	
Protection against TBE strains from separate geographical regions	04	Yes	No	
Vaccine manufacturer country origin	05	Europe	USA	
Environmentally friendly packaging (e.g., fully plastic-free)	06	Yes	No	
Where you can access the vaccine	07	Family doctor	Family doctor AND and SE: Specialist vaccination / travel clinic<br>CH and AUT: Pharmacist	

C2 Vaccine attribute stated importance

Base for question	All
Programming	Rating scale (1-7). Randomise order of rows<br><br>Keep information screen (from table 1) explanations as hyperlinks that can be clicked on in all proceeding questions
Question text	For the final task, we'd now like you to rate each of these TBE vaccine characteristics individually in terms of **their importance** when considering accepting or choosing a TBE vaccine for you or your family.
Respondent instruction	*Please rate the importance of each of the below TBE vaccine characteristics on a scale of 1-7 where 1=not at all important and 7=extremely important*<br><br>*Please click on the characteristic name if you would like to see its explanation again*
Rows	
01	Vaccine dosing schedule
02	Booster time interval
03	Ability to switch between different TBE vaccine brands
04	Protection against TBE strains from separate geographical regions
05	Vaccine manufacturer country origin
06	Environmentally friendly packaging (e.g., fully plastic-free)
07	Where you can access the vaccine
08	Effectiveness of the vaccine
09	Side effect frequency
10	Side effect severity
11	Recommendation of the vaccine by your doctor or healthcare professional
Columns	
01	Not at all important when considering a TBE vaccine
02	
03	
04	
05	
06	
07	Extremely important when considering a TBE vaccine

Thank for participation

End of Survey Sponsor Reveal

This market research and screening was carried out on behalf of Bavarian Nordic A/S (the "client"). While we will not disclose any of your personal data to our client, we are joint controllers with our client, and we have therefore included the client's privacy policy below.

If you have any questions about the processing of your personal data, including your rights, please contact us directly.

Market Research agency privacy policy: https://www.dayonestrategy.com/privacy

Bavarian Nordic's privacy policy:

PRIVACY POLICY –
MARKET RESEARCH (

CLOSE
