# Supplementary material for: What makes patients tick? Vaccine preferences against tick-borne encephalitis in four European countries
Source: BMC Infect Dis. 2024 Oct 13;24:1151. doi: 10.1186/s12879-024-10045-4 (PMC11472448; doi:10.1186/s12879-024-10045-4)
Supplement: Supplementary file 2 — Supplementary Material 2. [file 12879_2024_10045_MOESM2_ESM.docx]

**What makes patients tick? Vaccine preferences against tick-borne encephalitis in four European countries**

Charlotta Zacharias^1^; Ralph Torgler^2^; Jennifer Cummins^2^

^1^VaccinDirekt Sverige AB, Slussplan 7, 111 30 Stockholm Sweden

^2^Bavarian Nordic Switzerland AG, Grafenauweg 8, CH-6301 Zug, Switzerland

**Corresponding author:** Ralph Torgler

**Email address:** rato@bavarian-nordic.com

**Address:** Bavarian Nordic Switzerland AG, Grafenauweg 8, CH-6301 Zug, Switzerland

**Telephone number:** +41(0)762221288

**Supplementary figures/tables**


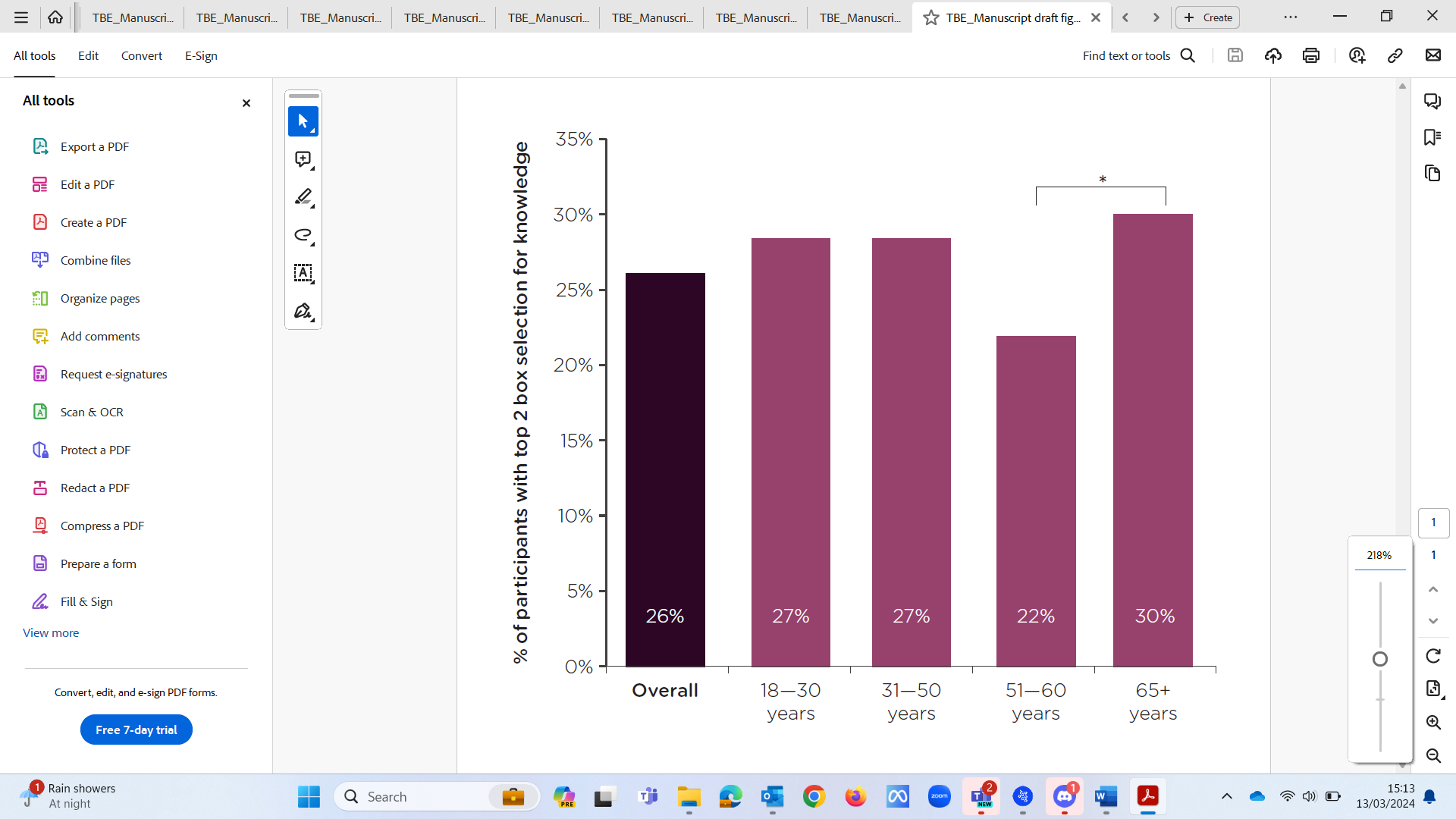
 **Supplementary Figure 1. Self-proclaimed vaccine knowledge in the overall study population, and by age subgroups**

Participants were asked: *On a scale of 1–7, with 1 being not at all knowledgeable and 7 being very knowledgeable, how knowledgeable would you say you are when it comes to vaccines?* Data are shown as the total % of participants with a top 2 box selection for knowledge (i.e., 6, knowledgeable; or 7, very knowledgeable).


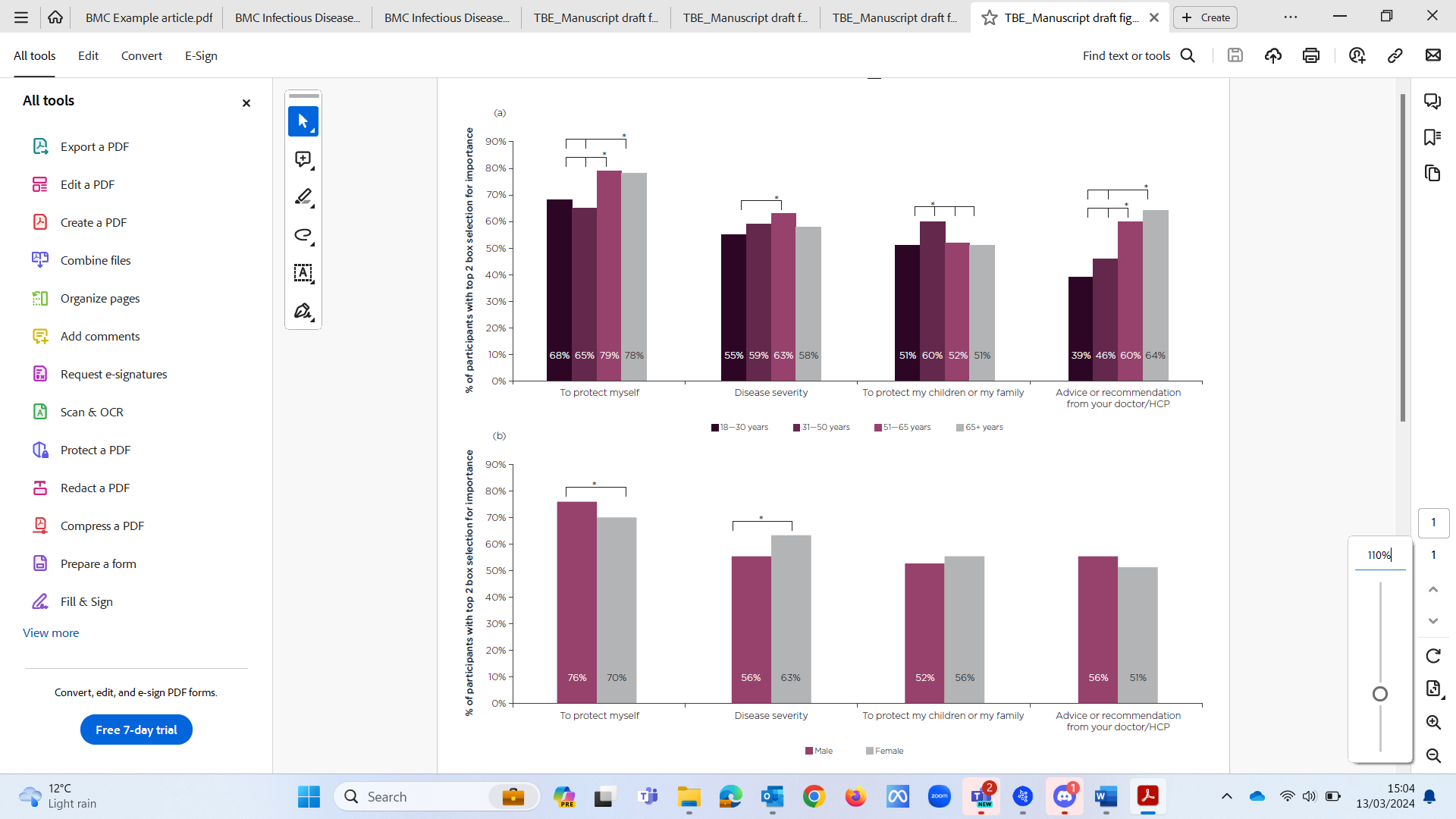
 **Supplementary Figure 2. Summary of motivating reasons for getting vaccinated ^a^, by age (a) or gender (b)**

*Denotes *P*-value ≤0.05. HCP, healthcare professional.
^a^Data are only shown for the four most commonly selected reasons as being among an individual’s top three selection. Participants were asked: *What are the most motivating reasons for getting yourself or your children vaccinated against infectious diseases? Please select your top three most motivating reasons.*

**Supplementary Table 1. Percentage of participants selecting a vaccine profile with each attribute level, by subgroup**

|  |  | **Endemic region** | | | | **Age** | | | | **TBE Vaccination** | | **Children  <18 years** | | **Endemic area** | |
| --- | --- | --- | --- | --- | --- | --- | --- | --- | --- | --- | --- | --- | --- | --- | --- |
|  | **Total** | **DE** | **SWE** | **CH** | **AUS** | **18–30** | **31–50** | **51–65** | **>65** | **Yes** | **No** | **Yes** | **No** | **Traveler** | **Resident** |
|  | n=1379 | n=500 | n=300 | n=301 | n=278 | n=218 | n=410 | n=400 | n=351 | n=342 | n=1037 | n=291 | n=1088 | n=320 | n=1059 |
| **SCHEDULE: Vaccine dosing schedule** |  |  |  |  |  |  |  |  |  |  |  |  |  |  |  |
| **Day 0/Day 7/Day 21** | 33% | 33% | 32% | 33% | 32% | 34% | 33% | 32% | 32% | 31% | 33% | 32% | 33% | 34% | 32% |
| **Day 0/1–3 Months/9–12 Months** | 34% | 34% | 35% | 34% | 35% | 34% | 34% | 35% | 34% | 35% | 34% | 34% | 34% | 35% | 34% |
| **Day 0/Day 14 /9–12 Months** | 33% | 32% | 33% | 33% | 34% | 32% | 33% | 33% | 34% | 34% | 33% | 33% | 33% | 31% | 33% |
| **BOOSTER: Booster time interval** |  |  |  |  |  |  |  |  |  |  |  |  |  |  |  |
| **3 years** | 20% | 20% | 23% | 20% | 19% | 23% | 19% | 19% | 22% | 20% | 20% | 22% | 20% | 22% | 20% |
| **5 years** | 29% | 28% | 31% | 28% | 30% | 31% | 28% | 28% | 30% | 31% | 28% | 29% | 29% | 29% | 29% |
| **10 years** | 51% | 53% | 46% | 53% | 51% | 46% | 54% | 53% | 48% | 50% | 51% | 49% | 51% | 50% | 51% |
| **SWITCH: Ability to switch between different TBE vaccine brands** |  |  |  |  |  |  |  |  |  |  |  |  |  |  |  |
| **Yes** | 55% | 54% | 58% | 54% | 56% | 56% | 55% | 54% | 56% | 56% | 55% | 55% | 55% | 54% | 55% |
| **No** | 45% | 46% | 43% | 47% | 45% | 45% | 45% | 47% | 44% | 44% | 45% | 45% | 45% | 46% | 45% |
| **PROTECTION: Protection against different TBE strains** |  |  |  |  |  |  |  |  |  |  |  |  |  |  |  |
| **Yes** | 68% | 68% | 70% | 69% | 67% | 67% | 68% | 70% | 68% | 68% | 68% | 67% | 69% | 68% | 68% |
| **No** | 32% | 32% | 30% | 31% | 33% | 33% | 32% | 30% | 33% | 32% | 32% | 33% | 31% | 32% | 32% |
| **ORIGIN: Vaccine manufacturer country of origin** |  |  |  |  |  |  |  |  |  |  |  |  |  |  |  |
| **Europe** | 61% | 61% | 58% | 60% | 63% | 60% | 60% | 62% | 61% | 58% | 62% | 59% | 61% | 61% | 61% |
| **USA** | 39% | 39% | 42% | 40% | 37% | 40% | 40% | 39% | 39% | 42% | 39% | 41% | 39% | 40% | 39% |

| **PACKAGING: Environmentally- friendly packaging  (e.g., fully plastic-free)** |  |  |  |  |  |  |  |  |  |  |  |  |  |  |  |
| --- | --- | --- | --- | --- | --- | --- | --- | --- | --- | --- | --- | --- | --- | --- | --- |
| **Yes** | 53% | 53% | 53% | 52% | 54% | 55% | 53% | 52% | 53% | 53% | 53% | 52% | 53% | 53% | 53% |
| **No** | 47% | 47% | 47% | 48% | 47% | 45% | 47% | 48% | 48% | 47% | 47% | 48% | 47% | 47% | 47% |
| **ACCESS: Where to access the vaccine** |  |  |  |  |  |  |  |  |  |  |  |  |  |  |  |
| **Family doctor** | 49% | 49% | 47% | 50% | 50% | 48% | 48% | 50% | 50% | 49% | 49% | 49% | 49% | 50% | 49% |
| **PCP + Pharmacist / specialist center** | 51% | 51% | 53% | 50% | 51% | 52% | 52% | 50% | 50% | 51% | 51% | 51% | 51% | 50% | 51% |

PCP, primary care physician; TBE, tick-borne encephalitis.

Data were taken from discrete-choice questions, where participants were asked to select their preferred TBE vaccine profile from three hypothetical vaccine profiles (repeated 12 times), assuming equal efficacy and tolerability across all profiles.
